# The intrinsic stiffness of human trabecular meshwork cells increases with senescence

**DOI:** 10.18632/oncotarget.3798

**Published:** 2015-04-12

**Authors:** Joshua T. Morgan, Vijay Krishna Raghunathan, Yow-Ren Chang, Christopher J. Murphy, Paul Russell

**Affiliations:** ^1^ Department of Surgical and Radiological Sciences, School of Veterinary Medicine, University of California, Davis, CA, USA; ^2^ Department of Ophthalmology &; Vision Science, School of Medicine, University of California, Davis, CA, USA

**Keywords:** trabecular meshwork, senescence, mechanobiology, cytoskeleton

## Abstract

Dysfunction of the human trabecular meshwork (HTM) plays a central role in the age-associated disease glaucoma, a leading cause of irreversible blindness. The etiology remains poorly understood but cellular senescence, increased stiffness of the tissue, and the expression of Wnt antagonists such as secreted frizzled related protein-1 (SFRP1) have been implicated. However, it is not known if senescence is causally linked to either stiffness or SFRP1 expression. In this study, we utilized *in vitro* HTM senescence to determine the effect on cellular stiffening and SFRP1 expression. Stiffness of cultured cells was measured using atomic force microscopy and the morphology of the cytoskeleton was determined using immunofluorescent analysis. SFRP1 expression was measured using qPCR and immunofluorescent analysis. Senescent cell stiffness increased 1.88±0.14 or 2.57±0.14 fold in the presence or absence of serum, respectively. This was accompanied by increased vimentin expression, stress fiber formation, and SFRP1 expression. In aggregate, these data demonstrate that senescence may be a causal factor in HTM stiffening and elevated SFRP1 expression, and contribute towards disease progression. These findings provide insight into the etiology of glaucoma and, more broadly, suggest a causal link between senescence and altered tissue biomechanics in aging-associated diseases.

## INTRODUCTION

Glaucoma is a family of irreversible blinding diseases that are projected to affect 79.6 million people worldwide by the year 2020 [1]. The most common form of glaucoma, primary open angle glaucoma, is an aging associated disease (AAD) often characterized by elevated intraocular pressure induced by increased outflow resistance of the aqueous humor [2]. The human trabecular meshwork (HTM), a complex three-dimensional structure comprised of cells, interwoven collagen beams and perforated sheets, is believed to provide the majority of outflow resistance in both normal and glaucomatous eyes [3-7]. HTM cells, depending on the region of the HTM, either form sheets covering extracellular matrix (ECM) structures or are scattered throughout the ECM [8-11]. What changes in the HTM resulting in increased resistance is poorly understood, but our recent study showed the HTM is ~20 fold stiffer in glaucoma [12], suggesting a prominent role of HTM mechanobiology. This tissue-scale stiffening is likely a result of biophysical changes to both the ECM and constituent cells, as structural changes to both the cytoskeleton [13, 14] and ECM [15-19] have long been associated with glaucoma.

Building upon these findings, further research has led to a growing body of evidence that these biophysical changes are not epiphenomena, but upstream of factors important in the progression of the disease. *In vitro* studies by our lab and others have shown that primary HTM cells have alterations in expression of genes associated with glaucoma, in biophysical properties, and in responsiveness to potential therapeutics when grown on hydrogels of varying stiffness [20-25]. Importantly, when cultured on hydrogels mimicking the stiffness of glaucomatous HTM, HTM cells increased expression of genes known to be associated with glaucoma progression [26-34], including myocilin [21, 23], secreted protein acidic and rich in cysteine (SPARC) [23], and secreted frizzled related protein-1 (SFRP1) [21]. These studies suggest a mechanism by which altered HTM mechanobiology reinforces the biological mediators of the glaucomatous phenotype. However, it remains unclear what processes induce stiffening.

A prime candidate for this process is cellular senescence, the irreversible arrest of cellular proliferation. Senescence is thought to contribute to many of the physiological changes associated with aging as well as AAD [35-40]. Induction of senescence generally occurs either due to telomere shortening after repeated mitosis (“replicative senescence”) or presentation of physiological stress (“stress-induced senescence”), although the distinction is blurry at best [35, 38, 41-47]. Both telomere shortening and cellular senescence are correlated with aging [48-52], and this is hypothesized as a prime driver of aging and associated diseases [53, 54]. Increased cell senescence is observed in the HTM of glaucoma patients [55], and glaucoma is likewise one among many AADs associated with increased rigidity of the tissue. Other AADs known to be associated with increased tissue stiffness include atherosclerosis [56-58], age-related macular degeneration [59-62], and cancer microenvironments [63-65]. Improved understanding of the causes of HTM stiffening in glaucoma will likely provide insight into other AADs as well.

Senescence is associated with increased expression of vimentin [66, 67] and filamentous actin (F-actin) [68-70], both of which are key determinants of cellular mechanics [71-74]. Both cytoskeletal elements are expressed in HTM cells [75-77], and altered F-actin morphology has been associated with HTM dysfunction [13, 14, 78-80]. Additionally, we have recently shown that exogenous SFRP1 induces pronounced and long-lasting stiffening of HTM cells [81]. SFRP1 has been shown to be necessary and sufficient for the induction of the senescent phenotype [82], suggesting SFRP1 induced stiffening may be related to senescence as well. In aggregate, there is strong support for a hypothesis of cellular senescence contributing to the glaucoma phenotype by increasing cellular stiffening associated with cytoskeletal changes. However, senescence has yet to be directly linked to HTM mechanobiology.

In this study, primary HTM cells were serially passaged until senescence and atomic force microscopy (AFM) was used to measure the intrinsic mechanical properties of senescent cells compared to normally proliferating controls. We found that stiffness was significantly increased in high passage HTM cells, and this was associated with increased staining of vimentin and F-actin. Further, SFRP1 expression was also elevated in senescent cultures. In aggregate, these results demonstrate HTM cellular senescence profoundly alters HTM mechanobiology and suggest a causal link between HTM cell senescence, altered cell mechanics and glaucoma progression.

## RESULTS

### Confirmation of senescence

**Figure 1:**
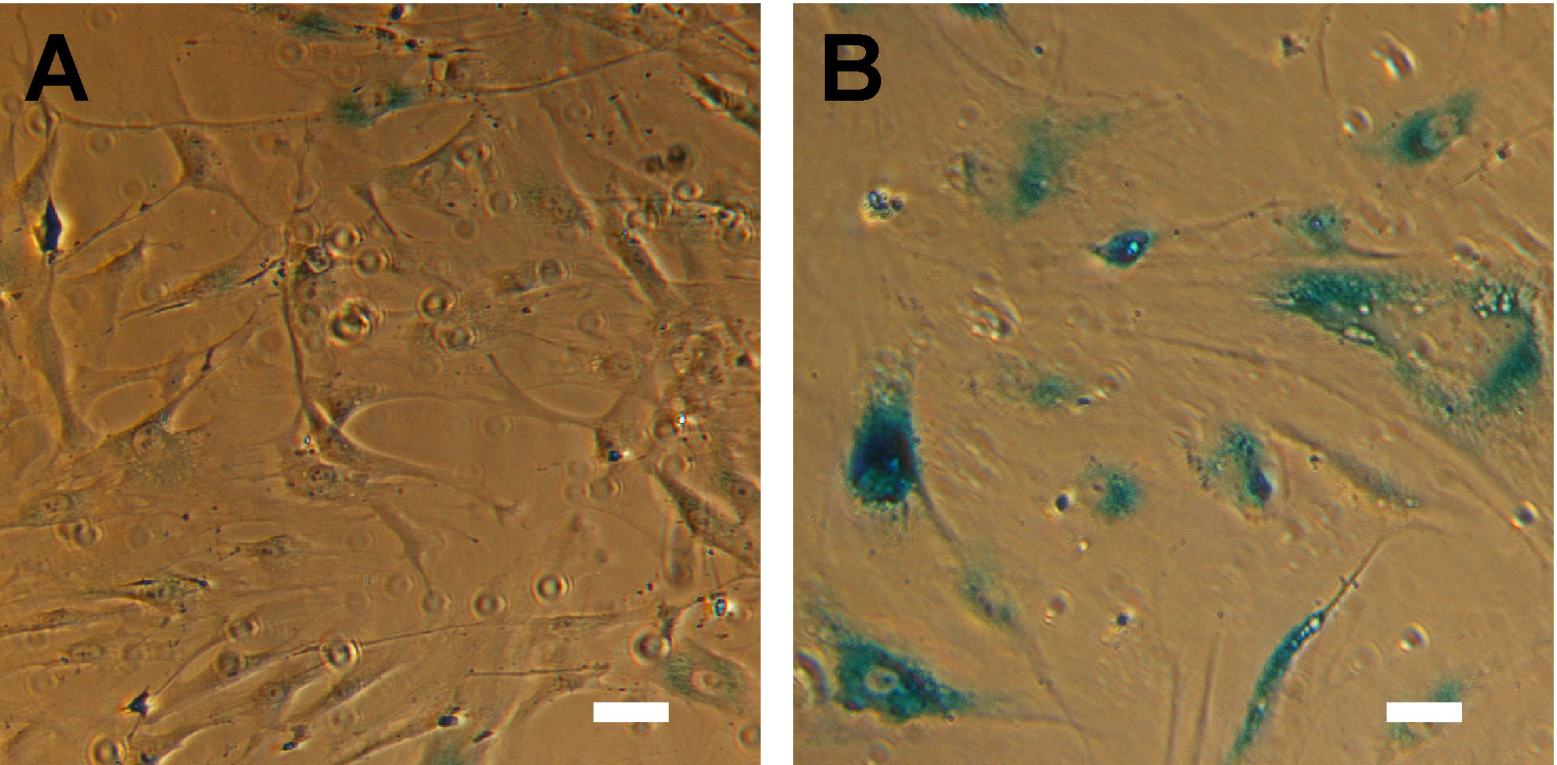
HTM cells exhibit hallmarks of senescence at advanced passage (**A**) HTM cells under routine culture are relatively small and elongated, and have minimal SAβGal activity. (**B**) After serial passaging, HTM cells assume a senescent phenotype included an enlarged, flattened morphology, and pronounced SAβGal activity. Images are of HTM728 (passages 5 and 7) and are representative of cells from other donors. Scale bars are 50 μm.

For all experiments, we serially passaged primary HTM cells until a complete loss of proliferative response was observed. Failure of proliferation was defined as having equal to or fewer viable cells one week after plating of a given passage. In these cultures, the cells took on an enlarged, flattened morphology, typical of senescent cells [83-88]. To confirm this method resulted in senescence, we plated cells of three donors (HTM553, HTM667, HTM631) on glass coverslips and assayed for senescence associated β-galactosidase (SAβGal) activity, a known marker of senescence [52, 89, 90]. In terminally passaged HTM cells, prominent blue staining indicates SAβGal activity which is minimal at earlier passages (Figure 1). Under these culture conditions, HTM cells typically senesced at passages 12-15, although cells isolated from some donors senesced earlier.

### Measurement of stiffness with increasing passage

**Figure 2:**
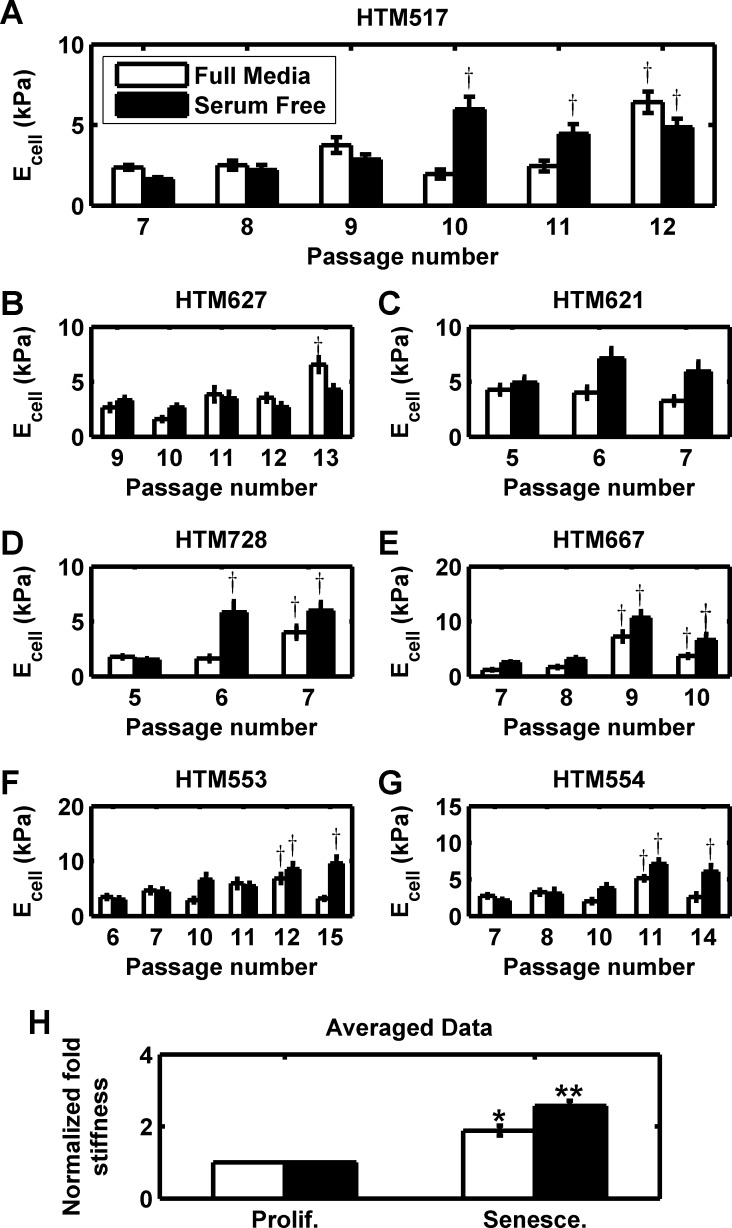
Intrinsic stiffness of HTM cells is increased in senescent populations (**A**) HTM cells from donor 517 underwent serial passaging and stiffness (E_cell_) was measured each passage for both full and serum free media conditions. At terminal passage, stiffness is substantially upregulated. (**B**-**G**) HTM cells from 6 other donors (627, 621, 728, 667, 553, and 554) similarly were serially passaged to senescence, and similarly exhibited stiffening at terminal passage. Data are mean±SEM. † *p* < 0.05 with respect to earliest passage. (**H**) For all 7 donors, the average elastic modulus at terminal passage (‘Senesce.’) was normalized to average elastic modulus at the earliest passage (‘Prolif.’). For each donor and each condition, 5-7 cells were each indented 5-7 times and averaged. Data are mean±SEM. * *p* < 0.05; ** *p* < 0.01.

Having established an *in vitro* model of HTM senescence, we turned to the primary objective of our study, determining if senescent HTM cells are intrinsically stiffer. We serially passaged primary HTM cells from 7 donors and measured cellular mechanics using AFM at each passage. Values of representative donor 517 are shown for passage 7 through senescence at passage 12 (Figure 2A). Minimal variation or serum dependence was observed at lower passages; the calculated elastic modulus of proliferating cells were similar to previously published values for HTM cells [25, 81, 91]. At terminal passage cells were substantially stiffer in full media, while the last three passages of cells in serum free media exhibited substantial stiffening. One way ANOVA and Bonferroni comparison to the earliest passage revealed these differences were significant to at least the *p* < 0.05 level, demonstrating a passage effect with cells from this donor. We performed similar experiments with cells derived from 6 other donors, observing similar results (Figure 2B-2G). Stiffness at or immediately before terminal passage was typically elevated in full media and/or serum free media, although the response in serum free media was more robust. Despite the apparent trend, there was substantial donor variability both in baseline stiffness and effect of passage. To control for donor-to-donor variability, we normalized the measurements of the senescent cells at terminal passage to proliferative cells at the earliest passage (Figure 2H). Senescent cell populations were 1.88±0.14 (n=7 donors; p < 0.05) or 2.57±0.14 (n=7 donors; *p* < 0.01) fold stiffer than proliferative controls in the presence or absence of serum, respectively.

### Staining of actin and vimentin with passage

**Figure 3:**
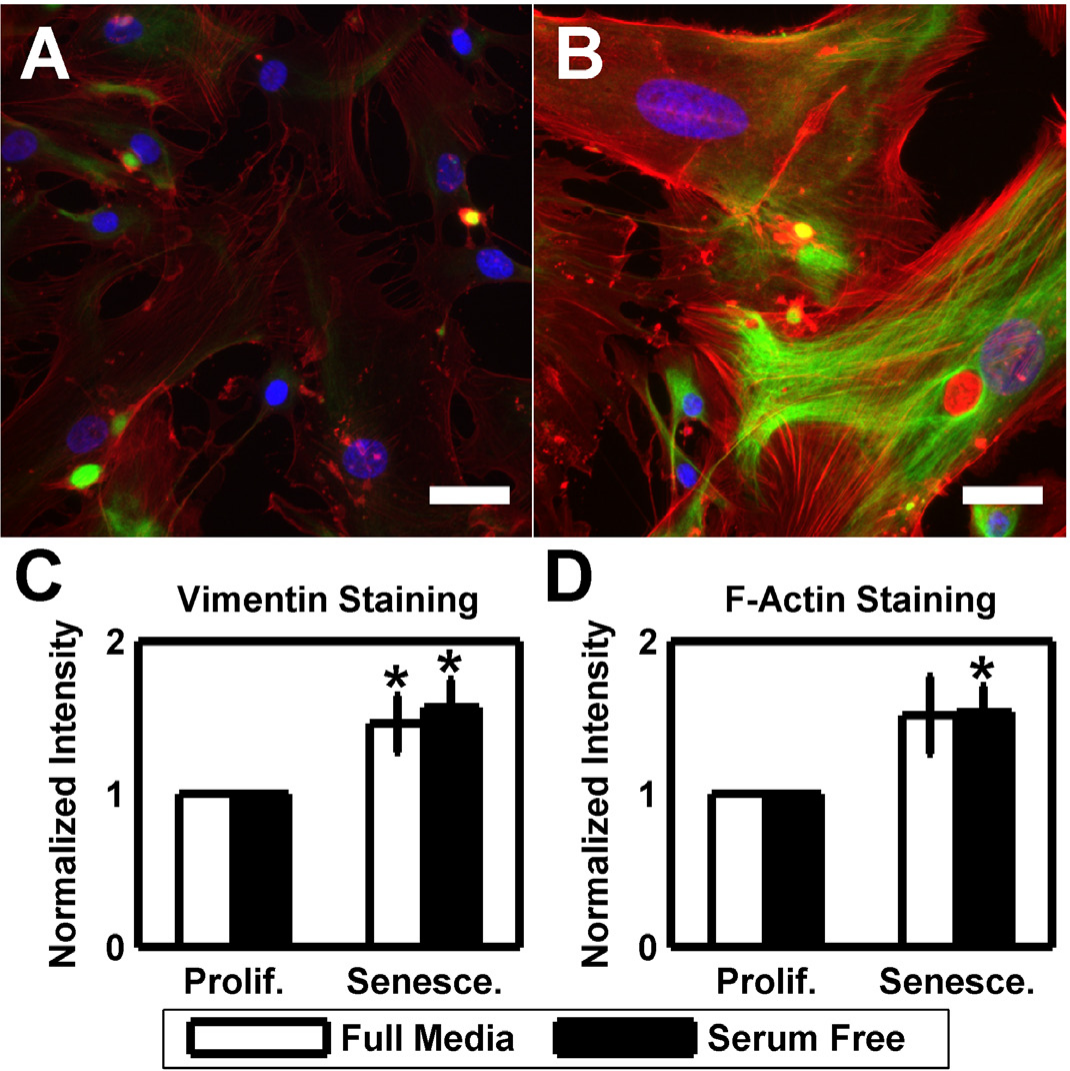
HTM cells exhibit more pronounced cytoskeleton at senescence (**A**) HTM cells under routine culture stain positively for both F-actin (red) and vimentin (green). DAPI used as nuclear counterstain (blue). (**B**) After serial passaging, HTM cells exhibit more pronounced stress fibers and brighter vimentin staining. Images are of HTM728 (passages 5 and 7) and are representative of cells from other donors. Scale bars are 50 μm. (**C**) Quantification of the average vimentin staining intensity reveals a significantly increased signal in senescent cultures when compared earlier passages (n=4). (**D**) Similar results are observed with average F-actin staining intensity, however, only serum free cultures demonstrate statistical significance (n=4). Data are mean±SEM. * *p* < 0.05.

As both intermediate filaments and the actin cytoskeleton have been linked to cell stiffness and senescence [66-74], we fixed and stained proliferative and senescent cultures for vimentin and F-actin (Figure 3A-3B). In proliferative cultures, vimentin was minimally expressed in the cell body but the expression was greatly increased in senescent cells. Similarly, proliferative HTM cultures exhibited numerous stress fibers as previously reported [14, 75, 79, 80], however, after senescence the stress fibers became more prominent. To quantify these changes, we averaged staining intensity of vimentin and F-actin for both senescent and proliferative cultures from 4 different donors (Figure 3C-3D). Senescent cultures exhibited significantly brighter staining intensity of vimentin, both in full (1.46±0.19 fold; *p* < 0.05) and serum free (1.57±0.18 fold; *p* < 0.05) media. Similar results were obtained with F-actin staining for full (1.52±0.25 fold; *p* = 0.086) and serum free (1.53±0.17 fold; *p* < 0.05) culture, although the results did not rise to significance in full media cultures.

### Expression of SFRP1 with passage

**Figure 4:**
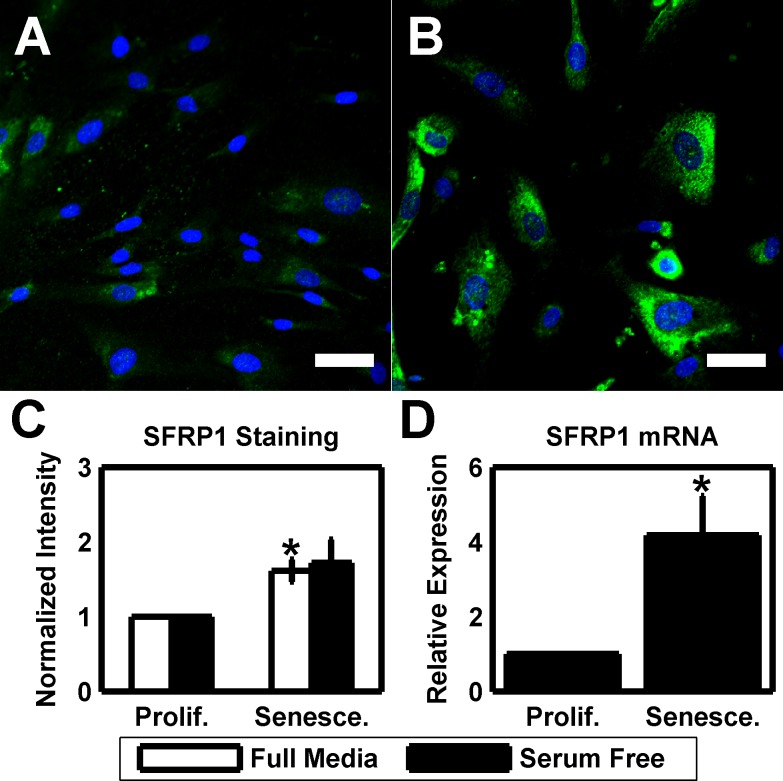
Elevated SFRP1 expression is a phenotype of HTM senescence (**A**) HTM cells under routine culture (HTM667; passage 6) stain positive for SFRP1 (green). DAPI is used as a nuclear counterstain. Scale bar is 50 μm. (**B**) After serial passaging, senescent HTM cells (HTM667; passage 10) exhibit a substantial increase in SFRP1 staining intensity. Images are of HTM667 (passages 6 and 10) and are representative of cells from other donors. Scale bars are 50 μm. (**C**) Quantification of the average SFRP1 staining intensity reveals a significantly increased signal in senescent cultures when compared earlier passages for full media cultures (n=3). While elevated, serum free cultures are not significant. (**D**) SFRP1 is elevated at the message level in serum free senescent cultures relative to earlier passages (n=3). Data are mean±SEM. * *p* < 0.05.

Finally, as we have previously linked exogenous SFRP1 to increased HTM cellular stiffness, we used immunofluorescence and qPCR to assay the expression of SFRP1 in proliferative and senescent cultures of 3 different donors. Proliferative HTM cultures stained positive for SFRP1 (Figure 4A), but this was greatly increased in senescent cells (Figure 4B). SFRP1 staining intensity was increased 1.6131±0.15 fold (p<0.05) in full media and 1.7211±0.31 fold (*p* = 0.080) in serum free cultures (Figure 4C). Additionally, we used qPCR to quantify SFRP1 mRNA expression. In serum free cultures of 3 different donors, SFRP1 was upregulated 4.18±1.07 fold (*p* < 0.05) with senescence.

## DISCUSSION

This study demonstrates senescence plays a prominent role in HTM mechanobiology. Utilizing serial passage of primary cells as an *in vitro* means of inducing senescence, this study demonstrates profound increases in cellular stiffness in senescent HTM cell populations. The increase occurred in the presence of serum but appeared more robust in serum free culture. This difference was an unexpected finding, and the role of serum signaling in senescence associated stiffening requires further study. The increase is correlated with, and likely related to, increases in vimentin, F-actin, and SFRP1 expression. Importantly, these data fill in crucial knowledge gaps in our understanding of HTM mechanobiology and build on previous work.

Our lab has shown that the HTM in glaucoma is ~20 fold stiffer [12], and available data suggests this is due to changes in both cytoskeletal dynamics and extracellular matrix [13-19]. Further, it is likely that these changes are coupled, as we have previously shown that HTM cells grown on stiffer matrices will have increased intrinsic stiffness [25] and elevated SFRP1 expression [21]. Despite the correlation of glaucoma with HTM stiffness, there is a paucity of data directly linking biological phenotypes of glaucoma with stiffening of the cells or matrix. Recently, we have shown that transforming growth factor β signaling, implicated in glaucoma, causes HTM cells to deposit a physically stiffer matrix with elevated SFRP1 secretion [92]. In a separate study, we demonstrated SFRP1, also associated with glaucoma, induces intrinsic stiffening of HTM cells [81]. Adding to these recent studies, we have now identified a third potential contributor to the overall stiffness of the HTM in glaucoma: cellular senescence.

It is instructive to consider the magnitude of the baseline HTM cell stiffness and the stiffening associated with senescence. As mentioned in the results, the baseline cellular stiffness is consistent with previous data on HTM cells [25, 81, 91] and is within the range previously reported for various cell types [93]. Further, the 2-3 fold change in HTM cellular stiffness with senescence is comparable to differences observed between metastatic and non-metastatic cancer cells [94-99], suggesting this difference could be coupled to dramatic functional changes. However, it is important to note that a 2-3 fold change is substantially smaller than the ~20 fold stiffening observed with glaucoma [12], suggesting senescence is but one of the factors contributing to HTM stiffening *in vivo*. The consequence that stiffening of cells has on ECM deposition and remodeling, particularly as it senesces, is poorly understood and warrants further investigations.

While the mechanisms underlying senescence-associated stiffening remain incompletely defined, it is likely cytoskeletal reorganization plays a key role. In numerous other studies, senescent cells or those from aged organisms exhibited marked changes to their cytoskeleton [66-70]. In this study, we stained for vimentin and F-actin, as both are known to be expressed in HTM [75-77] and relevant to cell stiffness [71-74]. Microtubules, while expressed in the HTM and altered in senescence [100-103], have been shown to have minimal impact on cellular mechanics [104, 105]. Another likely contributor, not explored in this study, is changes to the nuclear lamins, which contribute to nuclear stiffness [106, 107]. There is a well understood connectivity between the cytoskeleton and the lamina [108-110] and we feel it likely that the cytoskeletal alterations we have described are coupled with changes in lamin morphology. Indeed, the premature aging disease Hutchinson-Gilford progeria syndrome is linked to a splice variant of Lamin A, progerin [111, 112]. Progerin accumulation in cells leads to severe functional impairments [113-120], is associated with *in vitro* senescence [121], and has been identified in normal aging [122, 123]. As our studies utilize the nucleus as a consistent landmark for AFM indentation, the role of lamins is especially relevant. Future studies will investigate the role of lamin isoform expression in senescence associated stiffness.

We determined that SFRP1 is upregulated in HTM senescence (Figure 4), consistent with a previous report in different cell types [82]. SFRP1 is a potent inhibitor of Wnt signaling [124, 125], a key pathway that regulates numerous processes, including proliferation [126-129]. The findings of the current study serve to link findings from numerous previous reports by our lab and others. First, SFRP1 itself has been implicated in increased resistance to aqueous humor outflow in the HTM [32, 33], suggesting that senescence associated SFRP1 expression could directly hinder HTM function. Second, we have recently shown that SFRP1 induces HTM cell stiffening [81], providing a mechanism for a subpopulation of SFRP1 secreting cells to spread stiffening throughout the tissue. Third, HTM cells grown on hydrogels that mimic the stiffness of the glaucomatous HTM have increased stiffness as well as increased expression of SFRP1 [21, 25], suggesting that SFRP1 will be upregulated in the presence of tissue stiffening. Finally, high expression of SFRP1 has been shown to be sufficient to induce senescence in otherwise normal cells [82]. In aggregate, these data point to a feedback loop, where exposure to SFRP1 induces stiffening and senescence, which in turn induces further stiffening and SFRP1 expression/secretion to the extracellular milieu capable of affecting adjacent cells, compromising HTM function, and ultimately leading to glaucoma progression.

It is important to note that the above studies were performed on cells isolated from a range of donor ages (51-72 y.o.). It is possible that some of the observed donor variability is due to age, however, limited availability of tissue from a wide range of ages hinders the ability to properly explore these effects. As such, the above findings were not analyzed with respect to donor age due to the limited diversity of tissues.

Finally, while this study was performed specifically on HTM cells in the context of glaucoma, it potentially has broad applicability. Glaucoma exhibits many of the hallmarks of AADs, including fibrosis [15, 27, 130-133], oxidative stress [134-139], and loss of cellularity [55, 140-142]. It is quite possible the finding of increased stiffness with senescence is an aspect of many AADs. If so, further research into the causes and effects of this stiffening may identify novel therapeutic targets for treatment of a broad number of aging-associated diseases.

This study demonstrates a direct link between cellular senescence, intrinsic cell stiffening, and SFRP1 (a potent inhibitor of canonical Wnt signaling) expression in the HTM, all phenotypes associated with glaucoma. Taken with previous findings that SFRP1 can induce senescence and stiffening, these results suggest cellular senescence and resulting SFRP1 expression would induce further senescence and stiffening in neighboring cells, reinforcing and spreading the senescent phenotype and potentially leading to ocular hypertension and glaucoma. The identification of this feedback loop could have important ramifications for the design of effective therapeutics. Further, as glaucoma has many of the hallmarks of AADs, there is substantial potential that these findings may provide insight into other AADs.

## MATERIALS AND METHODS

### Culture of HTM cells and serial passaging

All experiments were performed in compliance with the Declaration of Helsinki. Primary HTM cells were isolated from eight donor corneoscleral rims (Saving Sight Eye Bank, St. Louis, MO) as described previously [143]. Cells derived from each donor are given a unique 3 digit identifier. For this study, HTM cells HTM517, HTM553, HTM554, HTM621, HTM627, HTM631, HTM667, and HTM728 were isolated from donors of age 51, 55, 55, 62, 62, 63, 66, and 72. HTM cells were cultured in DMEM/F12 (Hyclone, Logan, UT) with 10% fetal bovine serum (Atlanta Biologicals, Lawrenceville, GA) and 2 mM penicillin, streptomycin, amphotericin-B (Life Technologies, Carlsbad, CA). HTM cells were plated on glass coverslips at a 25,000 cells/cm^2^ and allowed to attach overnight. In order to isolate the influence of serum, cells were washed the following day and incubated in either serum-containing media (full media) or serum free media for three days before analysis.

Cells were routinely passaged at approximately 90% confluence. Cultures were continued at 1×10^6^ cells per 75 cm^2^ tissue culture flask. Cells were determined to be senescent when they failed to proliferate and exhibited a flattened, enlarged morphology. Failure of proliferation was determined by cell count (Cellometer Vision; Nexcelom, Lawrence, MA) as having equal to or fewer viable cells one week after plating of a given passage. A similar methodology has previously been successful at inducing a senescent phenotype in porcine trabecular meshwork cells [144]. This methodology was confirmed by the prominent expression of senescence associated β-galactosidase, assayed with a commercial kit (5 donors; Cell Signaling Technology, Danvers, MA). Cultures typically become predominantly senescent at passages 10 to 15, although 2 sets of donor cells senesced as early as passage 7.

### Atomic force microscopy analysis

Cell mechanics were determined as described previously using the Asylum MFP-3D-Bio AFM [25, 91]. Cells were rinsed in Hanks' Balanced Salt Solution (Hyclone, Logan, Utah), equilibrated on the AFM stage to minimize thermal drift, and indented in contact mode with silicon nitride cantilevers with square pyramidal tips (PNP-TR-50, Nano World, Switzerland). Prior to each experiment, the spring constant and deflection sensitivity of the cantilever were determined using thermal tuning and constant compliance methods. The elastic modulus (E) of each sample was obtained by fitting indentation force versus indentation depth to the Hertz model as shown in Eq. 1, where F is the force applied by indenter, α is the tip half angle (35^o^), ν is Poisson's ratio, and δ is indentation depth.

(1) $$F=\frac{2}{\pi }\frac{E\tan(\alpha )}{1-v^{2}}\delta^{2}$$

The Hertz model assumes that the samples were linearly elastic, homogenous, and infinitely thick. However, in the limit of small deformations, the Hertz model can be used for materials (such as cells) which are viscoelastic, heterogeneous, and finite [145]. Additionally, we assume the cells are incompressible (ν = 0.5), a good approximation for biological materials with high water content [146-149]. For each sample, approximately 5-7 cells were indented 5-7 times at 2 μm/s. All indentations were centered above the cell nucleus to minimize variability.

### Immunocytochemical analysis

Cells were washed in HBSS and fixed for 20 minutes in 4% formaldehyde and 0.25% Triton-X 100 (Fisher Scientific, Waltham, MA) in phosphate buffered saline (PBS) and blocked with 2% bovine serum albumin (Fisher), 0.2% gelatin (cold-fish; Sigma-Aldrich), 0.1% Tween-20 (Fisher) in PBS for 1 hr. Following blocking, cells were stained overnight using rabbit anti-SFRP1 (Abcam, Cambridge, UK) or rabbit anti-vimentin (Cell Signaling Technology, Danvers, MA) with a secondary of DyLight 488 conjugated goat anti-rabbit (Fisher). Nuclei and filamentous actin were counterstained with DAPI and phalloidin conjugated to Alexafluor 594, respectively. Cells were rinsed in PBS, mounted, and imaged on a Zeiss Axiovert 200 inverted microscope. All acquisition settings were kept constant for documentation of the same stain type (e.g. all phalloidin imaging utilized the same acquisition parameters). A minimum of 8 random fields were acquired for each case (approximately 100 cells). Cell borders were traced using semi-automated thresholding algorithm and mean gray scale intensity was averaged across the cell covered area across all images. For accurate quantification of the SFRP1 intensity, rolling ball background subtraction was employed. All analysis was conducted using custom image analysis scripts in MATLAB 2008b (Mathworks, Natick, MA). Thresholding and background subtraction parameters were manually checked for fidelity and applied uniformly across all images of a stain.

### Quantitative real-time polymerase chain reaction (qPCR)

Cells were cultured in 60 mm dishes under SF conditions as described above. After 3 days, the cells were lysed and mRNA was isolated using an RNeasy kit (Qiagen, Venlo, Netherlands) and the relative expression of SFRP1 was quantified from the mRNA by qPCR using SensiFAST Hi-ROX One-Step mastermix (Bioline, Taunton, MA) and Taqman primers (Hs00610060_m1; Life Technologies, Carlsbad, CA) using 60 ng of mRNA per reaction. Gene expression was normalized to that of the endogenous control, 18S rRNA (Hs99999901_s1; Life Technologies). Expression of the senescent cultures was determined relative to proliferative controls.

### Statistics

All experiments were with HTM cells isolated from multiple donors. The number of replicates is noted for each experimental set. For comparison AFM results within an individual donor cells, significance was assessed for full and serum free conditions separately using one-way ANOVA followed by Bonferroni's post-hoc test. Significant differences (p<0.05) from the earliest passage are indicated with †. For all other data, fold change of senescent cultures was determined in reference to control cells (proliferative cells of earlier passage) and significance was assessed using Student's t-test and significance denoted by ** = p<0.01, and * = p<0.05. All quantification is displayed as mean ± SEM and the number of biological replicates is noted.
